# Whole-genome analysis of *CGS*, *SAHH*, *SAMS* gene families in five Rosaceae species and their expression analysis in *Pyrus bretschneideri*

**DOI:** 10.7717/peerj.13086

**Published:** 2022-03-16

**Authors:** Yang Zhang, Decong Li, Xiaofeng Feng, Xinya Wang, Mengna Wang, Wenlong Han, Muhammad Aamir Manzoor, Guohui Li, Tianzhe Chen, Han Wang, Yongping Cai

**Affiliations:** 1 Anhui Agricultural University, Hefei, China; 2 West Anhui University, Luan, China

**Keywords:** CGS, SAHH, SAMS, Rosaceae, Expression analysis, Stone cell

## Abstract

Cystathionine γ-synthase (*CGS*), S-adenosyl-L-homocysteine hydrolase (*SAHH*), and S-adenosy-L-methionine synthetase (*SAMS*) play an important role in the regulation of plant growth, development, and secondary metabolism. In this study, a total of 6 *CGS*, 6 *SAHH*, and 28 *SAMS* genes were identified from five Rosaceae species (*Pyrus bretschneideri*, *Prunus persica*, *Prunus mume*, *Fragaria vesca*, and *Malus domestica*). The evolutionary relationship and microsynteny analysis in five Rosaceae species revealed that duplicated regions were conserved between three gene families (*CGS*, *SAHH*, *SAMS*). Moreover, the chromosomal locations, gene structures, conserved motifs, *cis*-elements, physicochemical properties, and Ka/Ks analysis were performed by using numerous bioinformatics tools. The expression of different organs showed that the *CGS*, *SAHH* and *SAMS* genes of pear have relatively high expression patterns in flowers and stems, except for *PbCGS1*. RNA-seq and qRT-PCR combined analysis showed that *PbSAMS1* may be involved in the regulation of pear stone cell development. In summary, this study provides the basic information of *CGS*, *SAHH* and *SAMS* genes in five Rosaceae species, further revealing the expression patterns in the pear fruit, which provides the theoretical basis for the regulation of pear stone cells.

## Introduction

Pear is an important fruit crop that belongs to the *Pyrus* genus in the Rosaceae family and has been cultivated for more than two thousand years in China (Xue et al., 2019). There are many types of pear fruits, which are rich in nutrients, and people love them. “Dangshan Su” Pear is originally produced in Dangshan County, Anhui Province, China, and is the most widely cultivated variety in China (Konarska, 2013). The fruit of pear is famous all over the world especially in China and become popular due to its high nutritional and medicinal value (Cao et al., 2018a; Wu et al., 2013). The content of stone cells is an important factor that affects the taste of pear. The less content of the stone cells increase the taste and quality of pear fruits, which is closely related to lignin (Jaime et al., 2015). Because stone cells are formed by the deposition of lignin on the secondary wall (Yan et al., 2014), it is important to reduce the synthesis of lignin.

**Figure 1:**
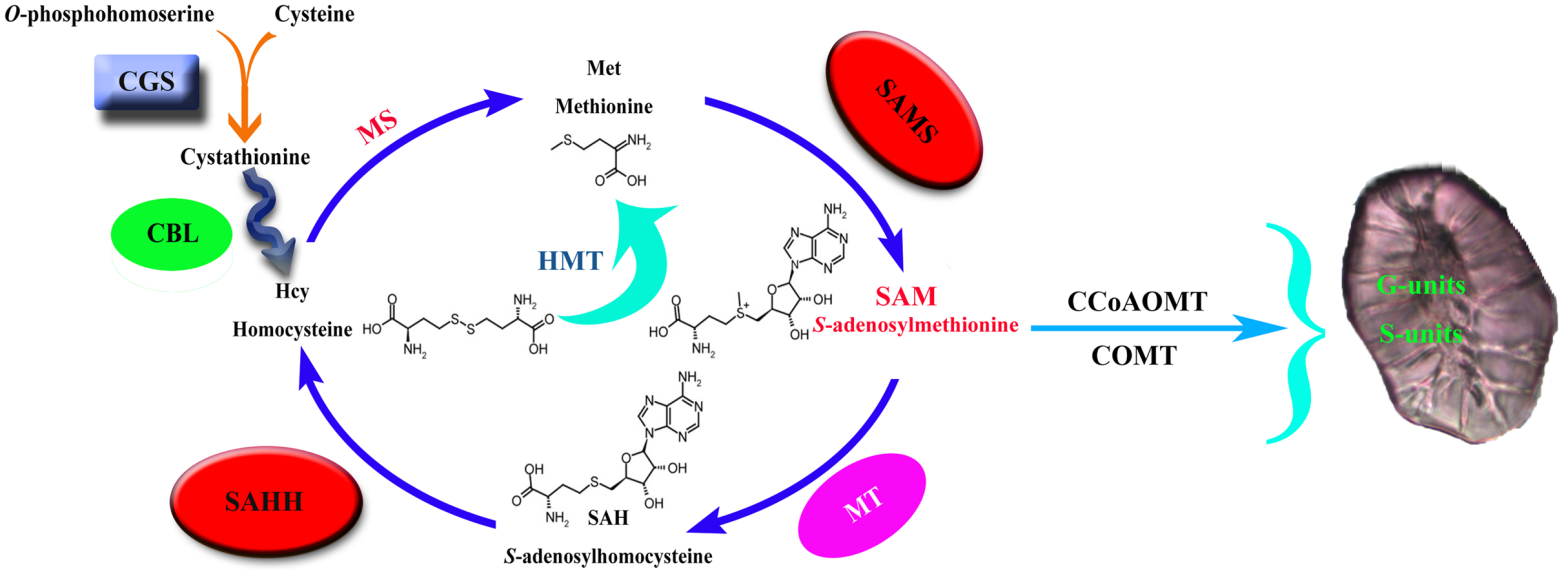
Lignin biosynthesis is controlled by carbon metabolism. CGS, cystathionine γ-synthase; CBL, cystathionine b-lyase; MS, methionine synthase; SAHH, S-adenosylhomocysteine hydrolase; HMT, homocysteine S-methyltransferase; SAMS, S-adenosylmethionine synthase; MT, methionine S-methyltransferase; CCoAOMT, caffeoyl CoA O-methyltransferase; COMT, caffeic acid O-methyltransferase.

Cystathionine γ-synthase (CGS) is the first step of methionine biosynthesis in higher plants and is a key regulatory step for methionine biosynthesis (Fig. 1). The O-phosphohomoserine (OPH) of aspartic acid (Asp) combines with the sulfhydryl group of cysteine to form cystathionine (Galili, Amir & Fernie, 2016). Threonine synthase (TS) competes with CGS for their common substrate OPH, which is metabolized into threonine (Thr) and isoleucine (Ile) (Thompson et al., 1982; Amir, Hacham & Galili, 2002). CGS and TS jointly control the biosynthesis of methionine. S-adenosyl-L-homocysteine hydrolase (SAHH) is the only eukaryotic enzyme that decomposes S-adenosyl-L-homocysteine (SAH), which is the key to maintaining SAH levels in plants. Sadenosylmethionine (SAM) is the methylation donor in the methylation reaction of all organisms. SAH is a byproduct of the reaction after the methylation donor is transferred to the acceptor, and it binds and competes with SAM, thereby inhibiting the activity of methyltransferase (Nguyen, Yablon & Chen, 2001; Luka, Mudd & Wagner, 2009). Therefore, SAH is a strong product inhibitor of SAM-dependent methyltransferase, which is hydrolyzed into homocysteine and adenylate by SAHH.

S-adenosy-L-methionine synthetase (SAMS) is a key enzyme in plant metabolism that catalyzes the synthesis of SAM from ATP and Met (Han et al., 2016). It has the functions of transmethylation, transthiolation and transamination (Belbahri et al., 2000; Finkelstein, 1990; Mato et al., 1997). Transmethylation mainly provides methyl groups for plants (Bottiglieri, Hyland & Reynolds, 1994). In the biosynthetic pathway of lignin, the methylation of G- and S-lignin monomers is mainly catalyzed by the two enzymes COMT and CCoAOMT (Humphreys, Hemm & Chapple, 1999; Osakabe, Tsao & Li, 1999; Ma & Xu, 2008), and the methyl group required for the reaction is provided by the synthesis of SAM (Bottiglieri, Hyland & Reynolds, 1994), so S-adenosylmethionine synthase is the key to lignin synthesis.

In plants, animals, and microorganisms, the transmethylation reaction is usually involved in the modification of almost all metabolites. In most methylation reactions, SAM is the methyl donor used by all organisms, while SAH is a by-product of the reaction after the transfer of the methyl donor to receptor (Luka, Mudd & Wagner, 2009; Moffat & Weretilnyk, 2010). SAH is a strong product inhibitor of SAM-dependent methyltransferase, which is hydrolyzed to homocysteine and adenosine by SAHH. SAHH is a key enzyme that maintains the methylation potential of cells (Tanaka et al., 1997; Miller et al., 1994). Inhibition of this enzyme will lead to increased accumulation of SAH, thereby inhibiting the methylation pathway through a feedback inhibition mechanism. CGS controls the synthesis of met. Overexpression of CGS can increase the level of met by 15 times and significantly increase the level of s-methylmethionine (SMM). SMM is considered to correspond to met (Baddiley & Jamieson, 1955; Eckert et al., 2009; Rao & Rossmann, 1973). SAM is also an effector of CGS post-transcriptional autoregulation, and the increase of SAM level down-regulates the level of CGS (Chiba et al., 2003). SAMS catalyzes the synthesis of SAM from ATP and Met and provides the necessary methyl groups for the lignin biosynthesis process. In summary, it can be seen that the three families of *CGS*, *SAHH* and *SAMS* are closely related.

Methyltetrahydrofolate reductase (MTHFR) and folic polyglutamate synthase (FPGS) can significantly affect the biosynthesis of lignin, which may help reveal a new regulatory mechanism of lignin biosynthesis (Li et al., 2015; Tang et al., 2014). MTHFR and FPGS participate in plant one-carbon (C1) metabolism, which is essential for the synthesis of Met, SAM, SAH, homocysteine (Hcy), and other methylated compounds in all organisms (Hanson & Roje, 2001). C1 metabolism generally includes tetrahydrofolate (THF) and the Met cycle. The former is responsible for the transfer of the C1 unit, and the latter is responsible for the synthesis and recovery of SAM (the activated form of Met). In the process of lignin biosynthesis, CCoAOMT and COMT need to use SAM as a cofactor to methylate the 3-OH and 5-OH on the aromatic ring, respectively, which leads to the biosynthesis of G and S lignin (Boerjan, Ralph & Baucher, 2003; Roje, 2006). In addition, many C1 metabolism genes show high expression levels in tissues with good lignification of plants, which means that there is potential coordination between C1 metabolism and lignin biosynthesis (Scully et al., 2018; Srivastava et al., 2015; Villalobos et al., 2012).

At present, the *CGS*, *SAHH* and *SAMS* genes of some species have been studied, such as *CGS1* in *Arabidopsis* (Chiba et al., 2003); *PvCGS* and *PvSAHH1* in switchgrass (Bai et al., 2018); *PtoSAHHA*, *PtoSAHHB* in poplar (Du et al., 2014); *SAHH1*-*SAHH2*, *SAMS1*-*SAMS4* (Yang et al., 2019) in *Arabidopsis*; *FvSAMS* (Kovacs et al., 2020) in strawberry and so on. Previous studies have been shown that overexpression of *AtCGS* leads to a significant increase in Met and SMM (Gakière et al., 2002); down-regulation of *PvSAHH1* will increase the level of SAH and reduce the ratio of SAM/SAH, thereby reducing the accumulation of lignin (Bai et al., 2018); the decrease of *AtSAMS3* can reduce the content of plant lignin (Shen, Li & Tarczynski, 2002). However, there are few reports on the bioinformatics and expression analysis related to the *CGS*, *SAHH* and *SAMS* gene families of pear (*Pyrus bretschneideri*). This study was conducted in the five Rosaceae species of pear, peach (*Prunus persica*), plum (*Prunus mume*), strawberry (*Fragaria vesca*), and apple (*Malus domestica*). Classification, conserved domains, chromosome location, microcollinearity, etc. were analyzed. Meanwhile, RNA-seq and qRT-PCR at different developmental stages of the *CGS*, *SAHH* and *SAMS* gene families of pear were analyzed to understand their expression patterns. The genes related to lignification in pear were screened out, and the study of five Rosaceae (*CGS*, *SAHH*, *SAMS* families) also laid the foundation for improving the quality of pear fruits.

## Materials and Methods

### Plant materials

The plant material collected from *Pyrus bretschneideri* Rehd. ‘Dangshan Su’, which is grown on horticulture orchards (Dangshan County, Anhui Province). The 40-year-old Dangshan crisp pear tree has been artificially pollinated. Uniform sized fruits were collected at eight-different development stages: 15 DAF (days after flowering), 39 DAF, 47 DAF, 55 DAF, 63 DAF, 79 DAF, 102 DAF, and 145 DAF. In addition, flowers, inflorescence stems, leaves, and buds samples were also collected in the same year. All of the samples were stored at −80 °C for further use.

### Identification and database search of *CGS*, *SAHH*, *SAMS* family members

The *Pyrus bretschneideri* and plum (*Prunus mume*) genomes were downloaded from the Genome Database For Rosaceae (https://www.rosaceae.org/), and the sequence information of strawberry (*Fragaria vesca*), peach (*Prunus persica*), and apple (*Malus domestica*) was downloaded from the Phytozome database (phytozome.jgi.doe.gov/pz/portal.html). The already known *Arabidopsis thaliana CGS*, *SAHH* and *SAMS* gene sequences were used as query (Chiba et al., 2003; Du et al., 2014; Shen, Li & Tarczynski, 2002). Secondly, the HMM (hidden Markov model) was searched through Pfam (http://pfam.xfam.org/) website to obtain the specific characteristic domain of *CGS*, *SAHH*, and *SAMS* families. Then the sequence was compared with BLASTP (E = <1.0E−10) in pear, strawberry, peach, apple, and plum genome database by using BioEdit software, and three gene family members were screened out among five Rosaceae species. Subsequently, SMART (http://smart.embl-heidelberg.de/) and the Pfam database were used to analyze the conserved domains of these genes, and the sequences without conserved domains and redundant sequences were removed (Cheng et al., 2018). Finally, the family members of the five Rosaceae species were identified.

### Comparative phylogeny analysis of *CGS*, *SAHH*, *SAMS* gene family

The amino acid and cds sequences of *CGS*, *SAHH* and *SAMS* genes were aligned by using clustalw tool in MEGA5.1 software, and the phylogenetic tree was constructed by Neighbor-Joining (N-J) method (bootstrap = 1,000; substitution model = p-distance) and Maximum Likelihood (ML) method (bootstrap = 1,000; substitution model = WAG). The evolutionary tree of these gene families was constructed between pear, strawberry, peach, apple, and plum.

### Analysis of genetic parameters in *CGS, SAHH, SAMS* gene family

The EXPASY (https://web.expasy.org/protparam/) online tool was used to predict isoelectric point (pI) and molecular weight (Mw). WoLF PSORT (https://www.genscript.com/wolf-psort.html) was used to predict subcellular localization.

### Conserved motifs and gene structure analysis of *CGS*, *SAHH*, *SAMS*

The online tool MEME (http://meme-suite.org/tools/meme), set the number of motifs to 20, the Minimum width, and Maximum width to 6 and 200. Finally use the TBTools (Chen et al., 2020) software to draw a conservative motif diagram. The gene structure was analyzed by GSDS (http://gsds.gao-lab.org/).

### Chromosomal locations and mode of duplication events

The genomic annotation data of five species were collected for chromosomal locations between three gene families. Finally, chromosomal locations were mapped with MapInspect software. Furthermore, the duplicate_gene_classifier tool of MCScanX was used to identify the duplication events in each species (Wang et al., 2012). In addition, the DnaSP 5.0 software was used to analyze the non-synonymous substitution rate (Ka), synonymous substitution rate (Ks) of duplicated genes.

### Promoter *cis*-acting elements and intraspecific microcollinearity analysis

The 2,000 bp promoter sequences upstream of the three gene family members were obtained from the pear genome database, and PlantCARE online webtool (http://bioinformatics.psb.ugent.be/webtools/plantcare/html/) was used to analyze the *cis*-acting elements on the promoter region. Then, TBtools (Chen et al., 2020) software was used to draw the intraspecies microcollinearity map.

### Expression patterns and transcriptomic analysis of *CGS*, *SAHH*, *SAMS* gene family

Download the transcriptome data of eight different developmental stages (15 DAF, 39 DAF, 47 DAF, 55 DAF, 63 DAF, 79 DAF, 102 DAF, and 145 DAF) of four pear varieties: Yali (*P. bretschneideri*), Starkrimson (*P. communis*), Nanguoli (*P. ussuriensis*) and Kuerlexiangli (*P. sinkiangensis*) fragrant pear from the NCBI database (Accession no. SRP070620). The expression profiles of *CGS*, *SAHH* and *SAMS* in the development of pear fruit were analyzed.

According to the RNA extraction kit of Tiangen Biotechnology Effective Company, the total RNA of Dangshansu pear fruit is extracted, and then cDNA is synthesized by the PrimeScript^RT^ reagent Kit with gDNA Eraser (Perfect Real Time) kit produced by the company. The Primer Premier 5.0 software was used to design specific primers. Detailed information about the primers is shown in Table S1. The relative expression levels of *CGS*, *SAHH* and *SAMS* genes in pear fruits were analyzed by qRT-PCR. The experiment uses the CFX96 fluorescent quantitative PCR instrument (Bio-Rad, Hercules, CA, USA), and the reaction system is 10 μL of SYBR® Premix Ex TaqTM II (2×) (TaKaRa, Kusatsu, Shiga, Japan), 2 μL of cDNA, 6.4 μL of water, and 0.8 μL of the upstream and downstream primers. This experiment was conducted with three biological and technical replication for each sample, and the relative expression of genes was calculated by 2^−ΔΔCT^ method (Livak & Schmittgen, 2001).

## Results

### Identification and amino acid characteristics of *CGS*, *SAHH*, *SAMS* family members

The members of *CGS* (6), *SAHH* (6), and *SAMS* (28) were identified from five Rosaceae species, which renamed in pear: *PbCGS1*, *PbSAHH1*-*PbSAHH2*, *PbSAMS1*-*PbSAMS6*; peach: *PpCGS1*, *PpSAHH*, *PpSAMS1*-*PpSAMS4*; plum: *PmCGS1*, *PmSAHH*, *PmSAMS1*-*PmSAMS4*; strawberry: *FvCGS1*, *FvSAHH*, *FvSAMS1*-*FvSAMS6*; and apple: *MdCGS1*-*MdCGS2*, *MdSAHH*, *MdSAMS1*-*MdSAMS8* (Table 1). The amino acid length of *CGS* members is between 412–618 aa, most of *SAHH* is 485 aa; only *PbSAHH2* is 502 aa; and the length of *SAMS* is between 253–448 aa, which has the biggest difference in length among apples. The molecular weights of *CGS* and *SAHH* are relatively high, mainly between 53.3–66.4 kDa, *PmCGS1* is relatively low, 45.9 kDa; *SAMS* is mainly in the range of 41.1–44.6 kDa, only *FvSAMS3*, *MdSAMS4*, and *MdSAMS6* individual members are different. Except for the isoelectric points of *PbCGS1*, *FvSAMS2*, and *MdCGS2* greater than 7, the other members are all less than 7, indicating that the amino acids are generally acidic. Prediction of subcellular location shows that four members of *CGS* gene family are found in the chloroplast, *PbCGS1* is in the chloroplast or endoplasmic reticulum, and *PmCGS1* may be in various positions of cells; 31 members of *SAHH* and *SAMS* gene family are in cytoplasmic, *MdSAMS4* is shown in the chloroplast or extracellular, while *FvSAMS2* and *MdSAMS6* are displayed in the chloroplast.

**Table 1:** Sequence attribute analysis of *CGS*, *SAHH* and *SAMS* gene family members.

Species	Sequence ID	Name	Protein				
			Length (aa)	Mw (kDa)	pI	Subcellular localization	Formula
**Pear**	Pbr038433.1	*PbCGS1*	618	66.4	9.09	chlo/E.R	C_2885_H_4628_N_804_O_849_S_20_
	Pbr005412.1	*PbSAHH1*	485	53.3	5.72	cyto	C_2358_H_3751_N_639_O_716_S_26_
	Pbr016485.1	*PbSAHH2*	502	55.1	6.22	cyto	C_2434_H_3874_N_666_O_732_S_28_
	Pbr008602.1	*PbSAMS1*	393	43.0	5.59	cyto	C_1906_H_2998_N_522_O_583_S_14_
	Pbr037756.1	*PbSAMS2*	393	43.0	5.59	cyto	C_1902_H_2999_N_521_O_584_S_15_
	Pbr026061.1	*PbSAMS3*	391	43.1	5.49	cyto	C_1909_H_3006_N_522_O_584_S_14_
	Pbr006707.1	*PbSAMS4*	394	43.2	5.56	cyto	C_1907_H_3005_N_525_O_587_S_15_
	Pbr020754.1	*PbSAMS5*	390	42.7	5.97	cyto	C_1897_H_3009_N_519_O_575_S_14_
	Pbr018549.1	*PbSAMS6*	379	41.2	5.53	cyto	C_1810_H_2860_N_502_O_565_S_15_
**Peach**	Prupe.3G264300.1	*PpCGS1*	522	56.0	6.51	chlo	C_2490_H_3981_N_677_O_744_S_21_
	Prupe.1G165200.1	*PpSAHH*	485	53.4	5.81	cyto	C_2363_H_3760_N_640_O_712_S_26_
	Prupe.1G107000.1	*PpSAMS1*	393	42.9	5.68	cyto	C_1899_H_3000_N_520_O_582_S_15_
	Prupe.3G004000.1	*PpSAMS2*	393	43.0	5.77	cyto	C_1899_H_2995_N_529_O_581_S_15_
	Prupe.6G306200.1	*PpSAMS3*	390	42.9	5.71	cyto	C_1904_H_3001_N_521_O_581_S_14_
	Prupe.7G128500.1	*PpSAMS4*	390	42.8	6.08	cyto	C_1895_H_3005_N_523_O_577_S_14_
**Plum**	Pm015918	*PmCGS1*	412	45.9	6.24	chlo/nucl/cyto/plas/extr/E.R	C_2052_H_3257_N_545_O_595_S_25_
	Pm007821	*PmSAHH*	485	53.5	5.81	cyto	C_2365_H_3764_N_640_O_712_S_29_
	Pm008469	*PmSAMS1*	393	42.9	5.68	cyto	C_1899_H_3000_N_520_O_582_S_15_
	Pm012890	*PmSAMS2*	393	43.0	5.60	cyto	C_1906_H_2999_N_521_O_583_S_14_
	Pm003235	*PmSAMS3*	390	42.9	5.79	cyto	C_1904_H_3003_N_521_O_579_S_15_
	Pm026349	*PmSAMS4*	390	42.9	6.08	cyto	C_1897_H_3009_N_523_O_577_S_15_
**Strawberry**	mrna04420	*FvCGS1*	545	58.4	6.57	chlo	C_2605_H_4148_N_706_O_775_S_21_
	mrna06564	*FvSAHH*	485	53.4	5.90	cyto	C_2367_H_3769_N_641_O_711_S_26_
	mrna09668	*FvSAMS1*	390	42.7	6.02	cyto	C_1890_H_2986_N_522_O_576_S_15_
	mrna13614	*FvSAMS2*	371	41.1	8.53	chlo	C_1856_H_2870_N_508_O_516_S_18_
	mrna13617	*FvSAMS3*	253	27.6	5.76	cyto	C_1228_H_1931_N_335_O_366_S_10_
	mrna22927	*FvSAMS4*	386	42.4	5.82	cyto	C_1881_H_2968_N_516_O_571_S_15_
	mrna22974	*FvSAMS5*	394	43.0	5.50	cyto	C_1901_H_3055_N_521_O_583_S_15_
	mrna24556	*FvSAMS6*	393	43.1	5.66	cyto	C_1909_H_3007_N_525_O_584_S_14_
**Apple**	MD17G1051900	*MdCGS1*	523	56.1	6.68	chlo	C_2501_H_3992_N_678_O_743_S_20_
	MD09G1054200	*MdCGS2*	547	58.9	7.62	chlo	C_2622_H_4188_N_714_O_776_S_23_
	MD13G1281900	*MdSAHH*	485	53.3	5.72	cyto	C_2355_H_3751_N_639_O_714_S_26_
	MD04G1091900	*MdSAMS1*	390	42.7	5.97	cyto	C_1896_H_2999_N_519_O_577_S_14_
	MD04G1187700	*MdSAMS2*	398	43.7	5.64	cyto	C_1930_H_3046_N_532_O_592_S_15_
	MD09G1292700	*MdSAMS3*	396	43.5	5.77	cyto	C_1922_H_3029_N_533_O_586_S_15_
	MD12G1109900	*MdSAMS4*	423	46.3	6.07	chlo/extr	C_2059_H_3269_N_559_O_622_S_15_
	MD12G1201100	*MdSAMS5*	395	43.6	5.66	cyto	C_1931_H_3048_N_530_O_587_S_15_
	MD13G1141700	*MdSAMS6*	448	49.1	6.20	chlo	C_2183_H_3449_N_597_O_658_S_17_
	MD16G1138300	*MdSAMS7*	393	43.0	5.59	cyto	C_1904_H_3004_N_518_O_583_S_15_
	MD17G1283400	*MdSAMS8*	406	44.6	5.74	cyto	C_1975_H_3103_N_541_O_600_S_17_

**Note:**

Chlo, Chloroplast; Cyto, Cytoplasmic; Nucl, Nucleus; plas, Plastosome; Extr, Extracellular; E.R., Endoplasmic reticulum.

### Phylogeny, classification, conservative motifs, and gene structure analysis of *CGS*, *SAHH*, *SAMS* genes

**Figure 2:**
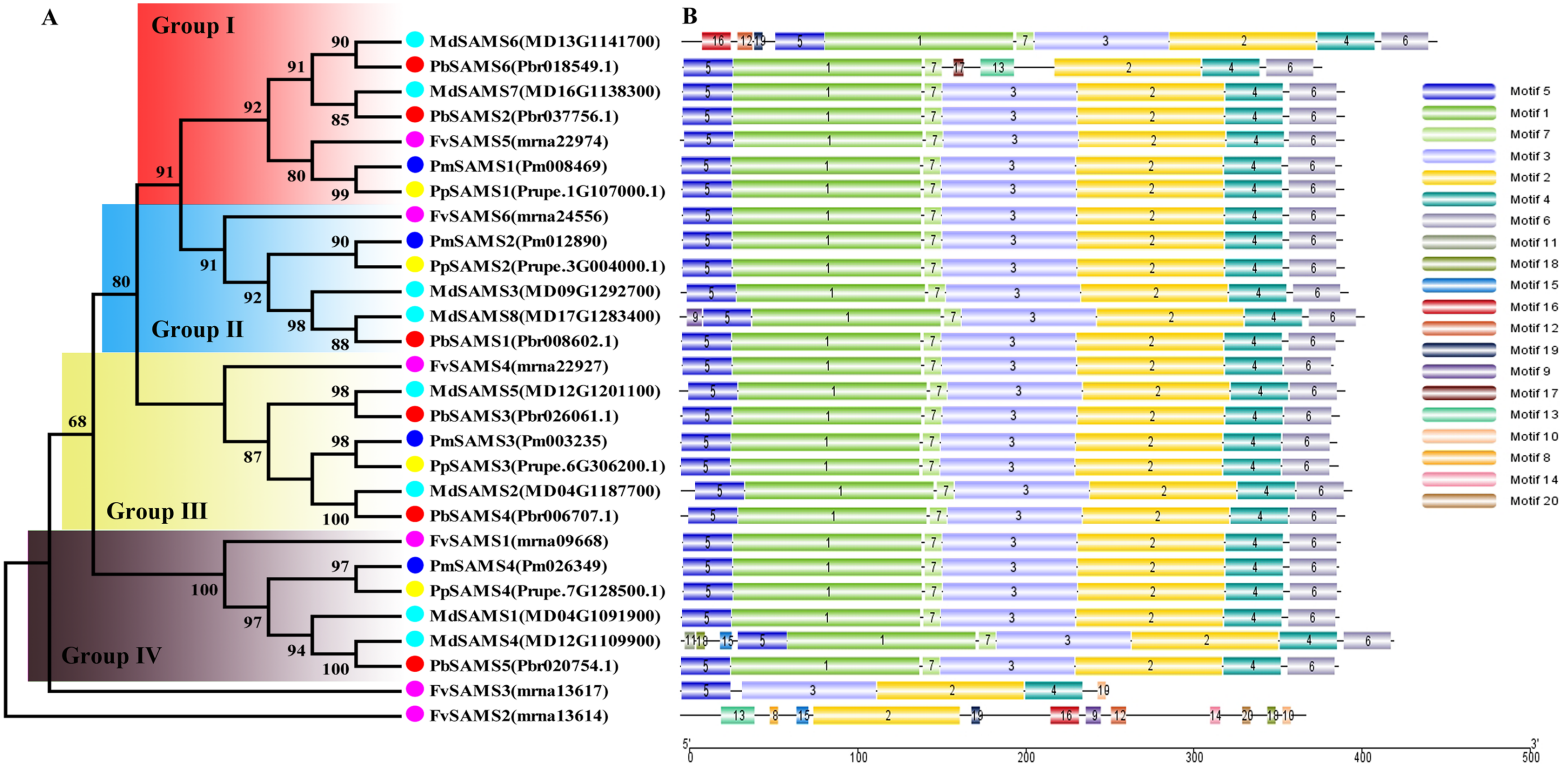
Systematic evolution and conserved motif analysis of *SAMS* genes family. (A) The phylogenetic tree of *SAMS* genes was conserved by the N-J method (It is constructed from amino acid sequences). (B) Conserved motif in *SAMS* proteins.

**Figure 3:**
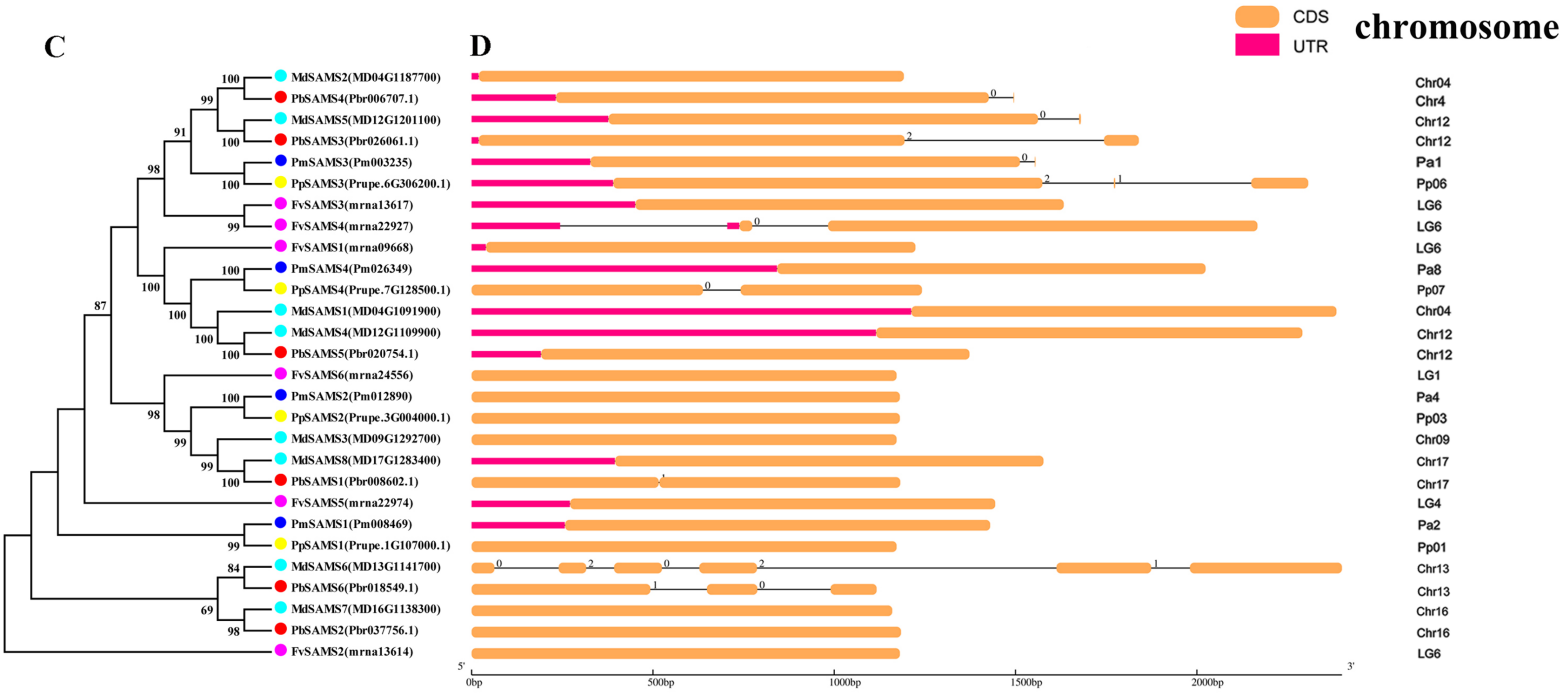
Systematic evolution and gene structures analysis of *SAMS* genes. (C) The phylogenetic tree of *SAMS* genes was conserved by the N-J method (It is constructed from cDNA sequences). (D) Exon-intron organization of *SAMS* genes.

**Figure 4:**
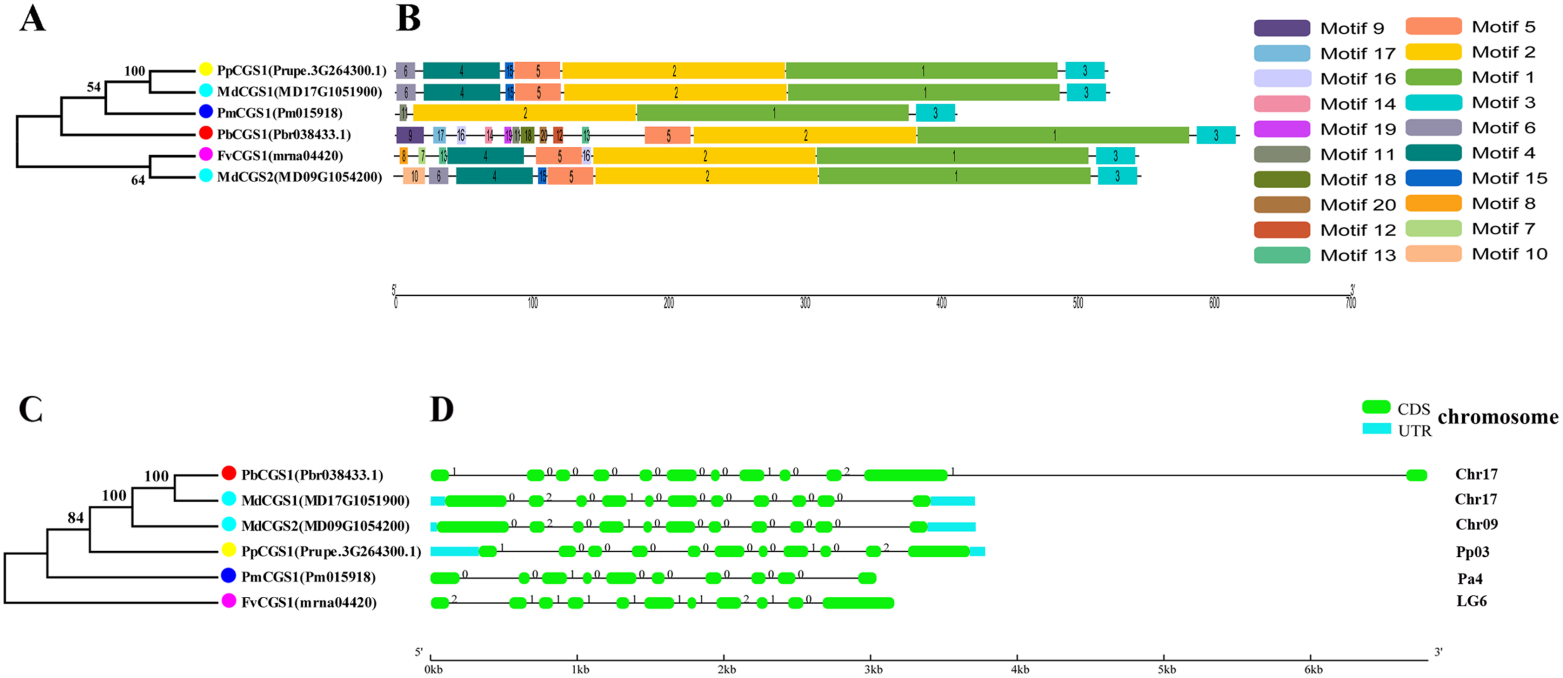
Systematic evolution, conserved motif and gene structures analysis of *CGS* genes family. (A) The phylogenetic tree of *CGS* genes was conserved by the N-J method (It is constructed from amino acid sequences). (B) Conserved motif in *CGS* proteins. (C) The phylogenetic tree of *CGS* genes was conserved by the N-J method (It is constructed from cDNA sequences). (D) Exon-intron organization of *CGS* genes.

**Figure 5:**
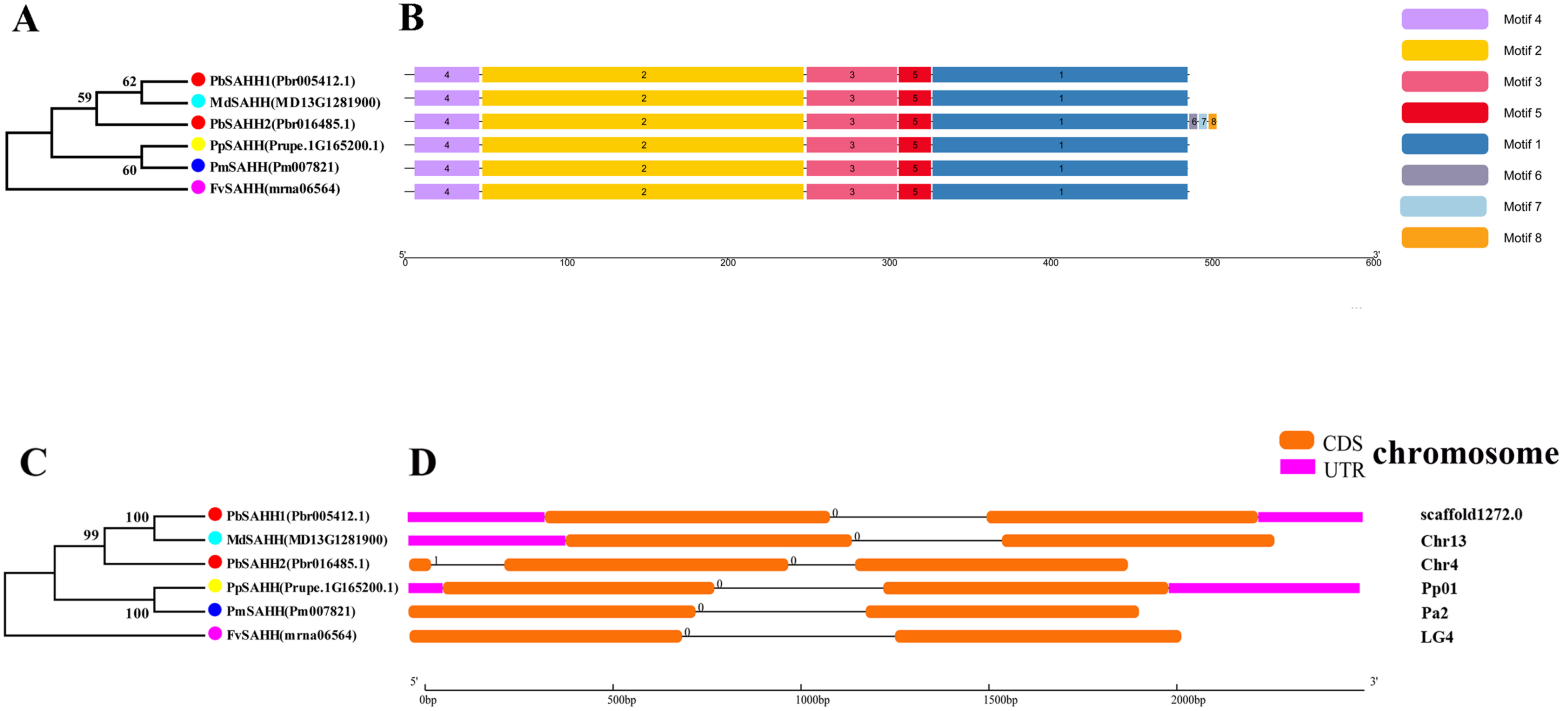
Systematic evolution, conserved motif and gene structures analysis of *SAHH* genes family. (A) The phylogenetic tree of *SAHH* genes was conserved by the N-J method (It is constructed from amino acid sequences). (B) Conserved motif in *SAHH* proteins. (C) The phylogenetic tree of *SAHH* genes was conserved by the N-J method (It is constructed from cDNA sequences). (D) Exon-intron organization of *SAHH* genes.

In this study, NJ method was used to construct the phylogenetic tree (Figs. 2A–5A). The conserved motif analysis and gene structure analysis corresponds to the phylogenetic tree constructed by amino acid sequence and cds sequence. The analysis of the *SAMS* gene family phylogenetic tree showed that the *SAMS* genes of the five species were mainly divided into four subfamilies, namely Group I, II, III, and IV (Fig. 2A). In addition, *FvSAMS2* and *FvSAMS3* form independent branches. The *SAMS* family members formed 10 gene pairs, and only three of them had high bootstrap values (≥99). Twenty motifs of the *SAMS* gene family were identified by using the MEME tool (Fig. 2B). Except for the two independent branches of *FvSAMS2* and *FvSAMS3*, the C-terminal conserved motifs of the four subfamilies include motif2, motif4, and motif6. Only *PbSAMS6* does not include motif3; except for the conservative motifs of *MdSAMS4*, *MdSAMS6*, and *MdSAMS8* at the N-terminal, most types and distributions are similar. The only *PbSAMS6* exists in motif17, and the only *FvSAMS2* exists in motif8 and motif20 indicating that they may have specific functions. The *SAMS* gene family is not all composed of CDS and UTR. Each subfamily and independent branch has UTR-free regions, and 16 of them have UTR regions (Fig. 3D). The C-terminal conserved motifs of the *CGS* gene family members are all consistent, including motif2, motif1, and motif3. Each member of the N-terminal contains a unique motif, which may contain some other functions; the UTR region and the non-UTR region each account for half (Fig. 4). Among the six members of the *SAHH* gene family, except for the *PbSAHH2* conserved motif, which contains motif6, motif7, and motif8, the remaining conserved motifs are the same. These motifs may give the member unique functions. UTR region and the non-UTR region also account for half (Fig. 5). Taken together, the conserved motif composition and gene structure characteristics of each family are very similar, which supports their close evolutionary relationship and the reliability of constructing evolutionary trees.

**Figure 6:**
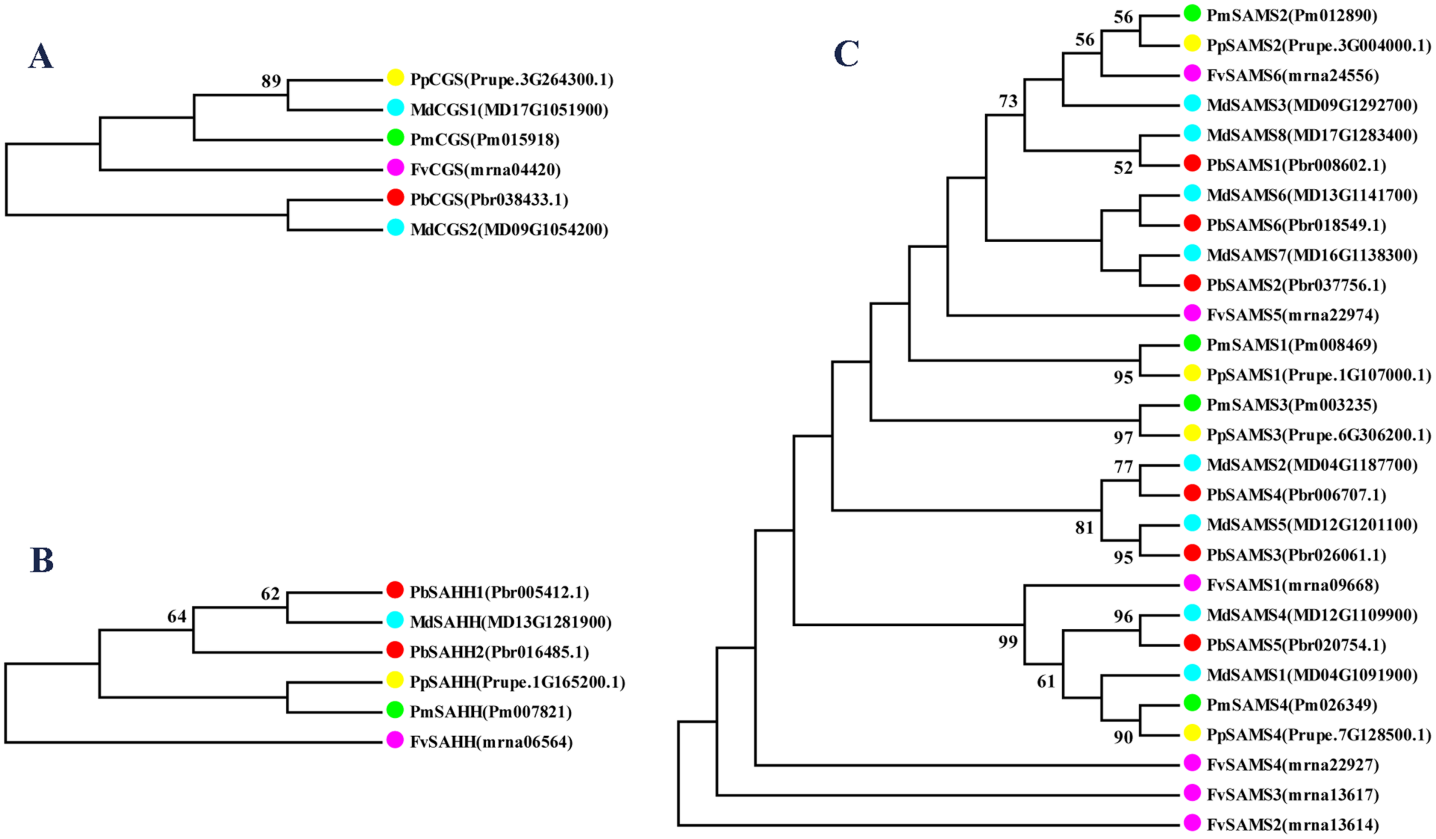
The maximum likelihood phylogenetic tree of *CGS*, *SAHH*, *SAMS* gene families. (A) The maximum likelihood phylogenetic tree of CGS gene family. (B) The maximum likelihood phylogenetic tree of *SAHH* gene family. (C) The maximum likelihood phylogenetic tree of *SAMS* gene family.

Meanwhile, we also constructed the ML phylogenetic tree (Fig. 6), and it is clear from the comparison between the two trees that the gene pairs formed due to high homology are basically consistent, indicating that the construction of the phylogenetic trees are accurate. Only in the *CGS* gene family, the homology of *PbCGS1*, *FvCGS1* and *MdCGS2* were not quite consistent in the two phylogenetic trees. Since the NJ phylogenetic tree is a distance method, which indicates the degree of similarity between sequences. While the amino acid sequences of *FvCGS1* and *MdCGS2* are closer, so they form a gene pair. ML phylogenetic tree indicates a series of features that change over time, and from an evolutionary perspective, it is possible that *PbCGS1* and *MdCGS2* are more closely related. Thus leading to a less consistent situation of these three genes.

### Characteristic domain analysis

Further analysis of the characteristic domains of five Rosaceae species from three families (Fig. S1) (Cheng et al., 2019). The result shows that the characteristic domain of *CGS* family members is Cys_Met_Meta_PP (PF01053). As can be seen from Fig. S1, according to Pfam’s annotation information (http://pfam.xfam.org/family/Cys_Met_Meta_PP), this domain uses pyridoxal phosphate (PLP) as a cofactor to participate in cysteine and methionine amino acid metabolism (Ferla & Patrick, 2014). PLP is the active form of vitamin B6. PLP is a versatile catalyst, as a coenzyme in many reactions, including decarboxylation, deamination, and transamination reactions (Hayashi, 1995; John, 1995; Kirsch & Eliot, 2004). The characteristic domain of *SAHH* family members is AdoHcyase_NAD (SM00997), according to Pfam’s annotation results (http://pfam.xfam.org/family/PF00670). S-adenosine-L-homocysteine acid hydrolase (AdoHcyase) is an activated methyl cycle enzyme responsible for the reversible hydration of S-adenosyl-L-homocysteine to adenosine and homocysteine. AdoHcyase is a ubiquitous enzyme that binds to and requires NAD^+^ as a cofactor (Sganga et al., 1992). The characteristic domains of *SAMS* family members mainly include S-AdoMet_synt_N (PF00438), S-AdoMet_synt_M (PF02772), and S-AdoMet_synt_C (PF02773). The three domains of S-adenosylmethionine synthetase have the same alpha + beta folding. S-adenosylmethionine synthase is an enzyme that catalyzes the formation of S-adenosylmethionine (AdoMet) from methionine and ATP (Horikawa et al., 1990). AdoMet is an important methyl donor for demethylation and a propylamine donor in polyamine biosynthesis. These characteristic domains indicate their important functions and further prove the accuracy of the identified members. However, the three members of *FvSAMS2*, *FvSAMS3*, and *PbSAMS6* are missing one domain, which may change their functions.

### Chromosome location and gene duplication analysis of *CGS*, *SAHH*, *SAMS* family members

**Figure 7:**
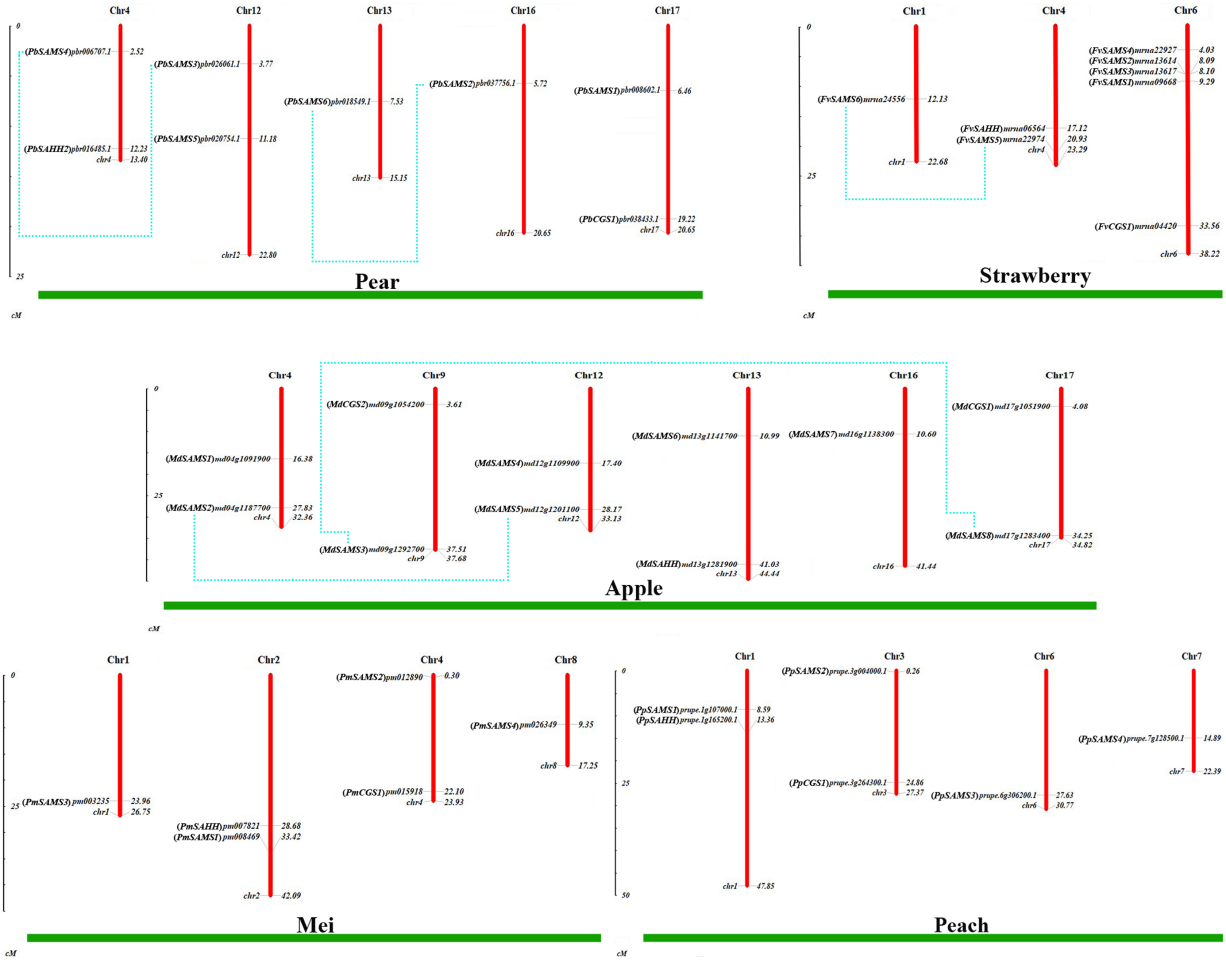
Chromosomal locations and gene duplication of five Rosaceae species. The species name is marked above the green line. duplicated gene pairs are connected with dotted lines.

To understand the distribution of *CGS*, *SAHH* and *SAMS* families on chromosomes, the chromosome locations of five species were analyzed (Fig. 7). Except for *PbSAHH1*, all members of *CGS*, *SAHH* and *SAMS* gene family members were located on chromosomes. In *Pyrus bretschneideri*, eight members are distributed on chromosomes 4, 12, 13, 16, and 17. In *Fragaria vesca*, eight members were mainly distributed on chromosome 6, and the rest on chromosomes 1 and 4. The 11 members of *Malus domestica* were evenly distributed on chromosomes 4, 9, 12, 13, 16, and 17. In *Prunus mume*, six members are distributed on chromosomes 1, 2, 4, and 8. In *Prunus persica*, six members are distributed on chromosomes 1, 3, 6, and 7.

**Figure 8:**
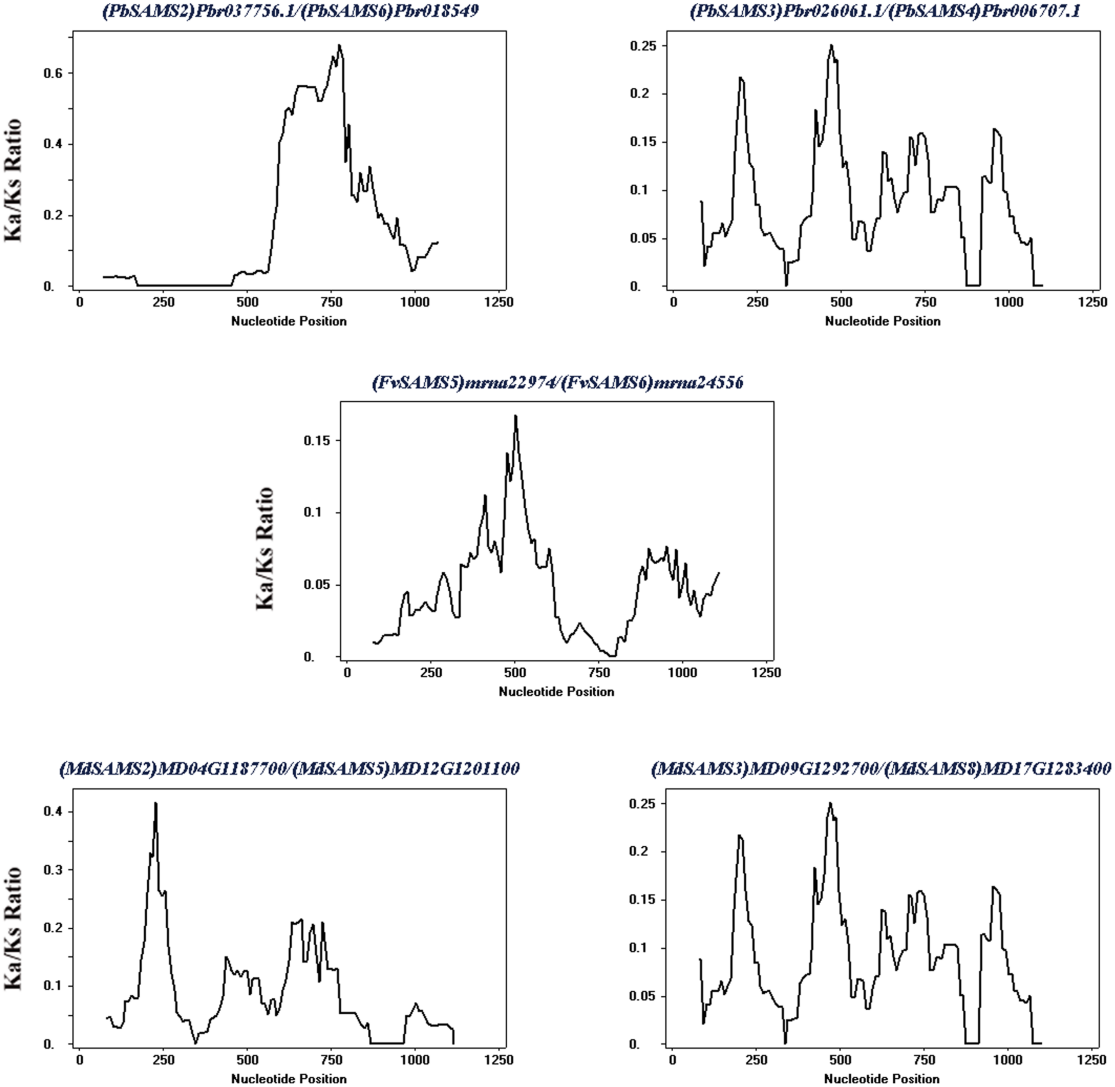
Sliding window analysis of duplicated genes. At the top of each sliding window is a duplicate gene pair.

Subsequently, the evolutionary relationship analysis of these three gene families and the chromosome locations, it is found that five gene pairs have different modes of duplication events. They are all shown in Fig. 7 and each pair is connected together with a dotted line. The analysis showed that they have a high percentage of segmental duplication, indicating that the amplification of *SAMS* gene family through a segmental duplication event. According to Table 2 and Fig. 8, Ka is less than Ks and the peak value is not 1. Also we used functional motifs for analysis and comparison (Table S2), and found that the calculated Ka and Ks values of the two results are very close, and the results are consistent. It indicates that the gene has purification and selection effects, and the structure and function are not seriously affected.

**Table 2:** Analysis of gene duplication events of *CGS*, *SAHH* and *SAMS* gene family members (They use whole cDNA sequences).

Duplicated genes	Ka	Ks	Ka/Ks	Duplicated type
(*PbSAMS2*) *Pbr037756.1/*(*PbSAMS6*) *Pbr018549.1*	0.0389	0.2679	0.1452	Segmental duplication
(*PbSAMS3*) *Pbr026061.1/*(*PbSAMS4*) *Pbr006707.1*	0.0170	0.2044	0.0832	Segmental duplication
(*FvSAMS5*) *mrna22974/*(*FvSAMS6*) *mrna24556*	0.0452	1.0571	0.04276	Segmental duplication
(*MdSAMS2*) *MD04G1187700/*(*MdSAMS5*) *MD12G1201100*	0.0180	0.2512	0.0717	Segmental duplication
(*MdSAMS3*) *MD09G1292700/*(*MdSAMS8*) *MD17G1283400*	0.0078	0.2149	0.0363	Segmental duplication

### Intraspecies microcollinearity

**Figure 9:**
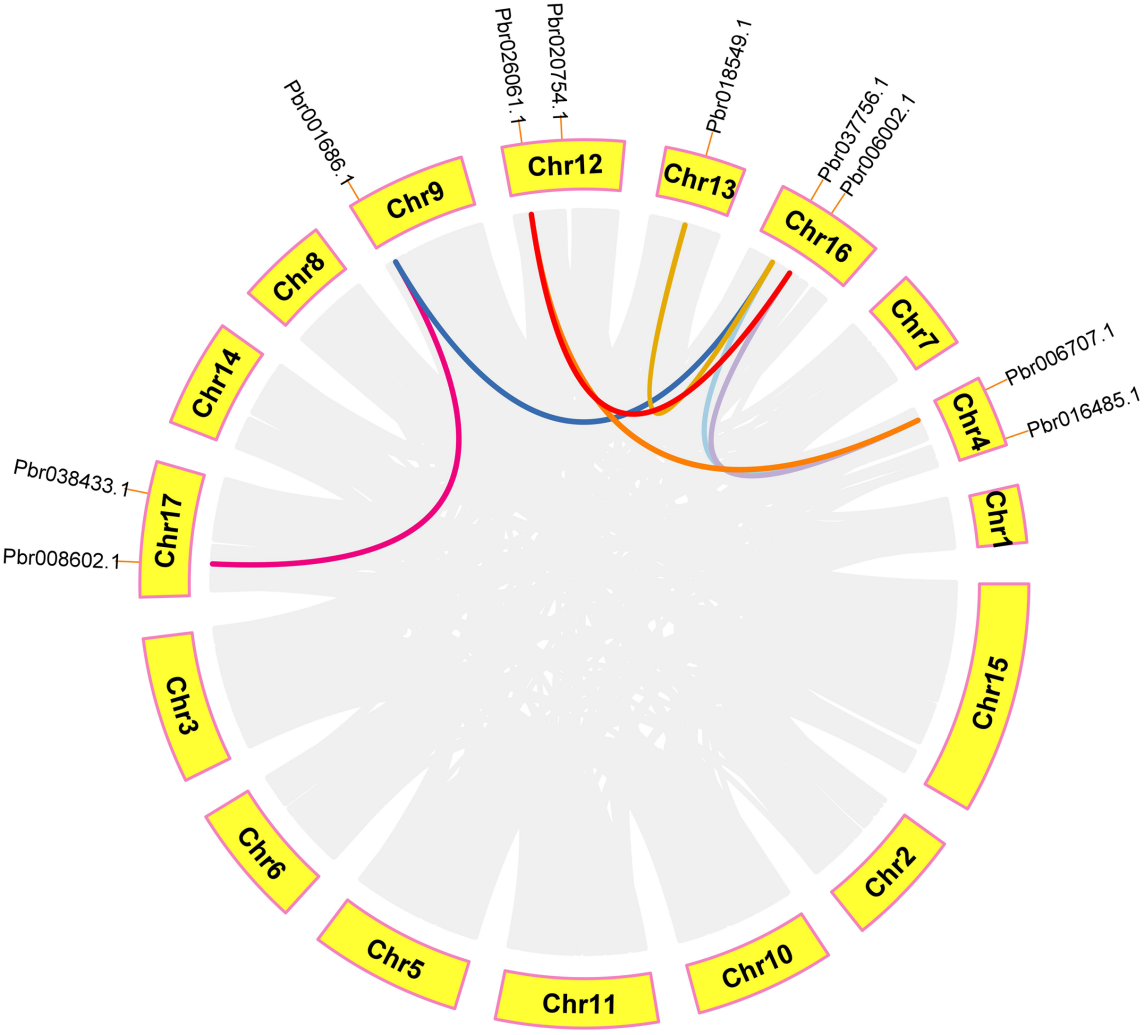
Analysis of microcollinearity within pear species. Each collinear gene pair is connected with colored lines.

In order to further analyze the collinearity relationship of *CGS*, *SAHH* and *SAMS* gene families in pear, and we identified seven pairs of collinearity genes in a pear that have high homology, indicating that may be due to similar functions (Fig. 9). Respectively: *Pbr008602.1*/*Pbr001686.1*, *Pbr018549.1*/*Pbr037756.1*, *Pbr026061.1*/*Pbr006002.1*, *Pbr026061.1*/*Pbr006707.1*, *Pbr018549.1*/*Pbr037756.1*, *Pbr037756.1*/*Pbr006707.1*, *Pbr006002.1*/*Pbr006707.1*.

### Identification of *cis*-acting elements analysis

**Figure 10:**
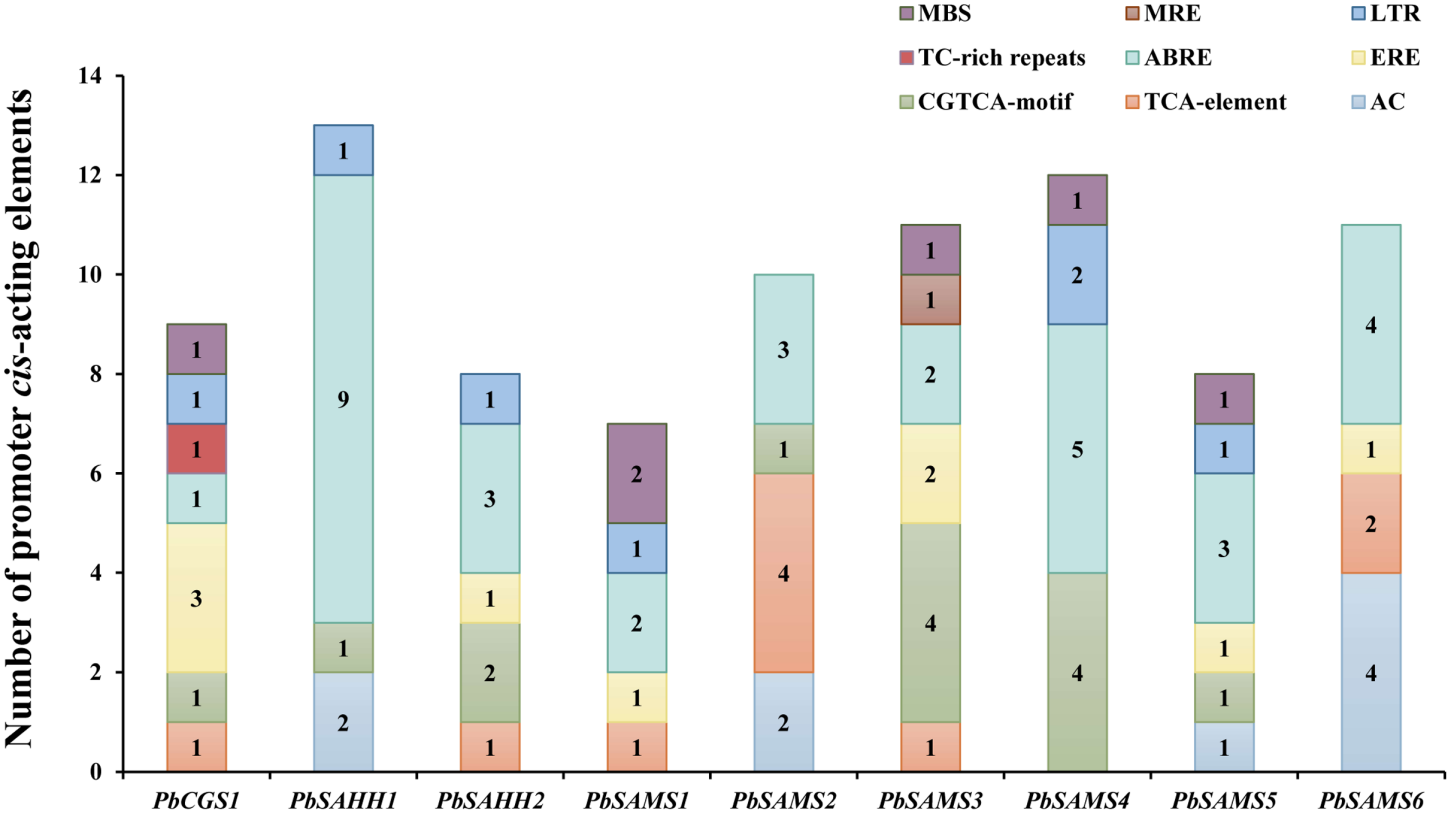
Analysis of *cis*-acting elements of *PbCGS*, *PbSAHH* and *PbSAMS*. Potential *cis*-elements in the 5′ regulatory sequences of the nine genes.

To better understand the promoter characteristics of members of the *CGS*, *SAHH* and *SAMS* gene families in pear, we analyzed their *cis*-acting elements (Fig. 10). Among these nine genes, only *PbSAMS3* contains MRE (light response element), indicating that only the expression of this member may be regulated by light. These members also contain elements related to defense and abiotic stress, such as TC-rich repeats (defense and stress response), MBS (drought induction), LTR (low-temperature response). Except for *PbSAMS2* and *PbSAMS6*, all other members contained at least one element of MBS or LTR, indicating that *PbCGS*, *PbSAHH*, *PbSAMS* are closely related to pear drought and low-temperature stress. We also found many CGTCA-motif, TCA-element, ABRE elements and ERE elements related to MeJA, SA, ABA and ethylene hormone response. All of the nine members contained ABRE elements, a total of 32, which are the most abundant *cis*-acting elements, indicating that they have the most extensive response to abscisic acid. The *cis*-acting elements TCA-element and ERE related to SA and ethylene were identified in six different members. CGTCA-motif, a *cis*-acting element related to MeJA, was identified among the seven members, a total of 14, which is the second most abundant *cis*-acting element. In addition to these elements, we also found AC elements that bind to MYB, which are related to lignin synthesis (Cao et al., 2016). These results indicate that *PbCGS*, *PbSAHH* and *PbSAMS* not only play a role in the synthesis of lignin, but also participate in the light response process, abiotic stress, and hormone response.

### Comparative phylogenetic analysis and function prediction

**Figure 11:**
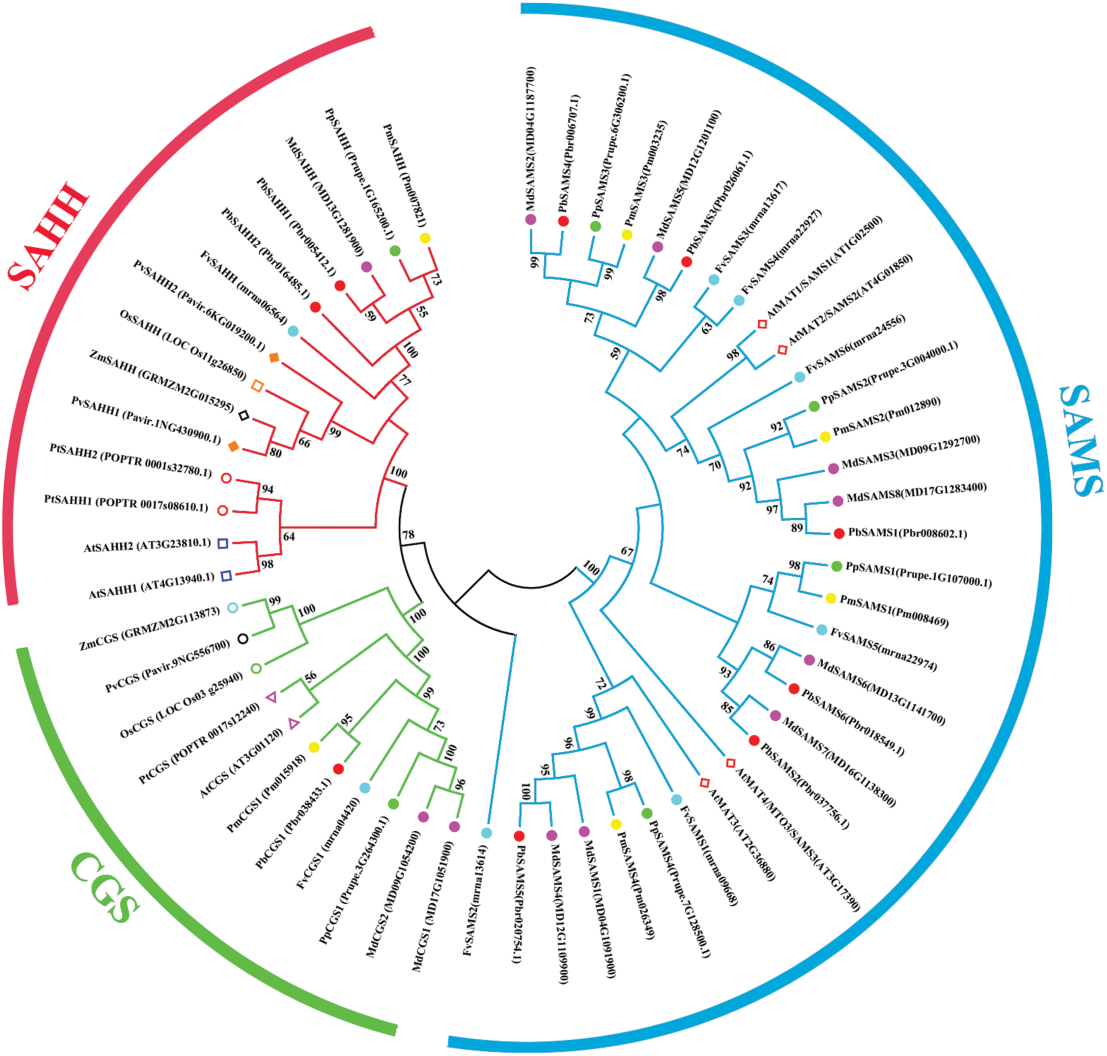
Phylogenetic tree analysis of *CGS*, *SAHH* and *SAMS* species in different plants. The interspecific phylogenetic tree is composed of three families: *CGS*, *SAHH*, and *SAMS*. The green, red and blue lines represent the three families, respectively.

In this study, 10 species of *CGS*, *SAHH*, and six species of *SAMS* were constructed to form an interspecies complex phylogenetic tree (Fig. 11), and the members of the *CGS*, *SAHH* and *SAMS* families in pear were studied. We constructed 10 species of *CGS*, *SAHH*, and six species of *SAMS* to form an interspecific complex phylogenetic tree, and studied the members of the *CGS*, *SAHH* and *SAMS* families in pear. *PbCGS1* and *AtCGS* are very close to the branch of the phylogenetic tree. In previous studies, it was found that overexpression of *AtCGS* significantly increased Met and SMM. *CGS* is the rate-limiting enzyme in this metabolic pathway (Gakière et al., 2002). *PbCGS1* may also have a similar role.

Numerous studies have found that down-regulating *PvSAHH1* will increase SAH levels and reduce the SAM/SAH ratio, thereby reducing the accumulation of lignin (Bai et al., 2018). *PvSAHH1* is in the same sub-branch as *PbSAHH1* and *PbSAHH2*, indicating that these two genes may have this function. However, *PbSAHH1* and *PbSAHH2* are not in the same gene pair, and their functions may be slightly different. Four members of *the SAMS* family have been identified in *Arabidopsis thaliana*. Among these four members, *AtSAMS3* shows different functions from other members. The reduction of *AtSAMS3* results in the decrease of lignin content in plants, indicating that *AtSAMS3* plays a role in lignin biosynthesis (Shen, Li & Tarczynski, 2002). The *SAMS* members in pear are evenly distributed near *AtSAMS3*, such as *PbSAMS1*, *PbSAMS2*, *PbSAMS5*, etc., indicating that these members may participate in lignin biosynthesis.

### Expression profile analysis of *PbCGS*, *PbSAHH* and *PbSAMS* genes

**Figure 12:**
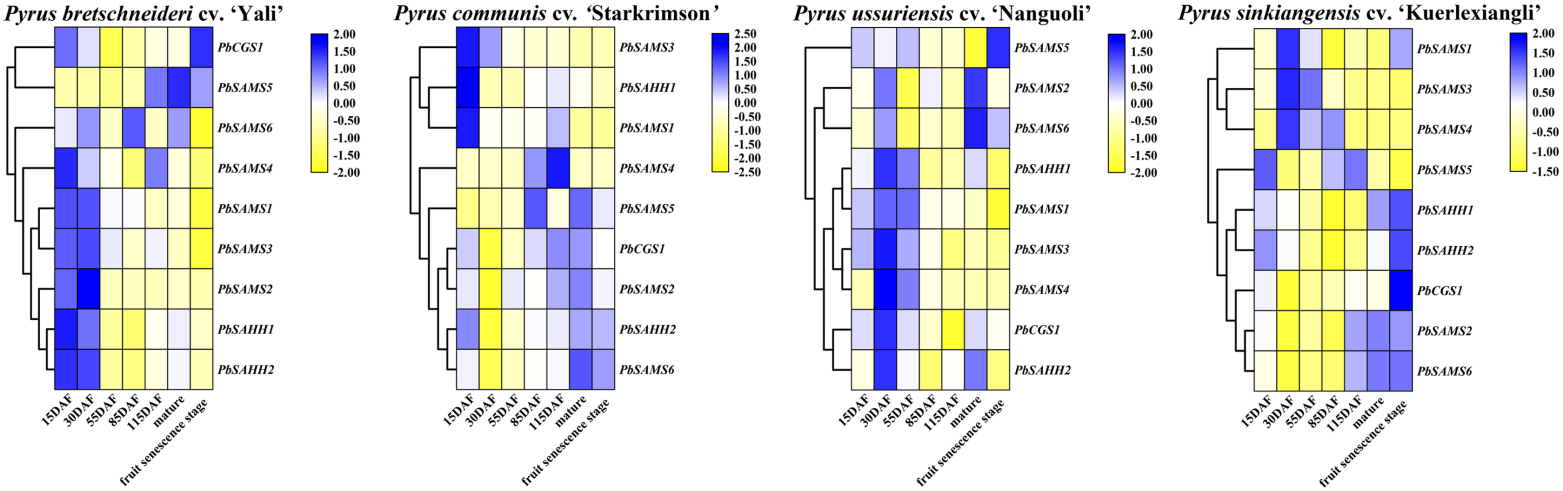
Expression profile analysis of *PbCGS*, *PbSAHH* and *PbSAMS* in the development of four pear fruits. The heat map is generated by the TBtools software based on the RNA-seq database. Gene transcription levels are indicated by different colors on the heat map.

*PbCGS1* expressed higher in the first three periods (15 DAF, 30 DAF, and 55 DAF) in Nanguoli, but lower in the other three pear. *PbSAMS2* was also only expressed higher in the first three periods in Yali (Fig. 12). The expression levels of *PbSAHH1* and *PbSAHH2* were lower in the first three periods of Kuerlexiangli, but higher in the other three pear. The expression of *PbSAMS4* was only low in the first three stages of Starkrimson. The expression levels of *PbSAMS5* and *PbSAMS6* in the corresponding period are not consistent with the changing trend of lignin synthesis and stone cell development, indicating that there is no connection with it. These identical genes have different expression in different varieties of pear fruit. By promoter analysis predicts that these members are closely related to drought stress. It may be different due to the differences between geographical, cultivation and environmental conditions, and it is inconsistent in pear fruit (Arzani et al., 2008). The expression levels of *PbSAMS1* and *PbSAMS3* in Yali, Starkrimson, Nanguoli and Kuerlexiangli were higher in the first three stages, but lower in the four stages after flowering. This is consistent with the trend of lignin synthesis and stone cell development (Cai et al., 2010; Cao et al., 2016; Cheng et al., 2016). These two members may be involved in lignin synthesis and stone cell development.

### Analysis of *PbCGS*, *PbSAHH* and *PbSAMS* genes expression patterns in Dangshan Su Pear

**Figure 13:**
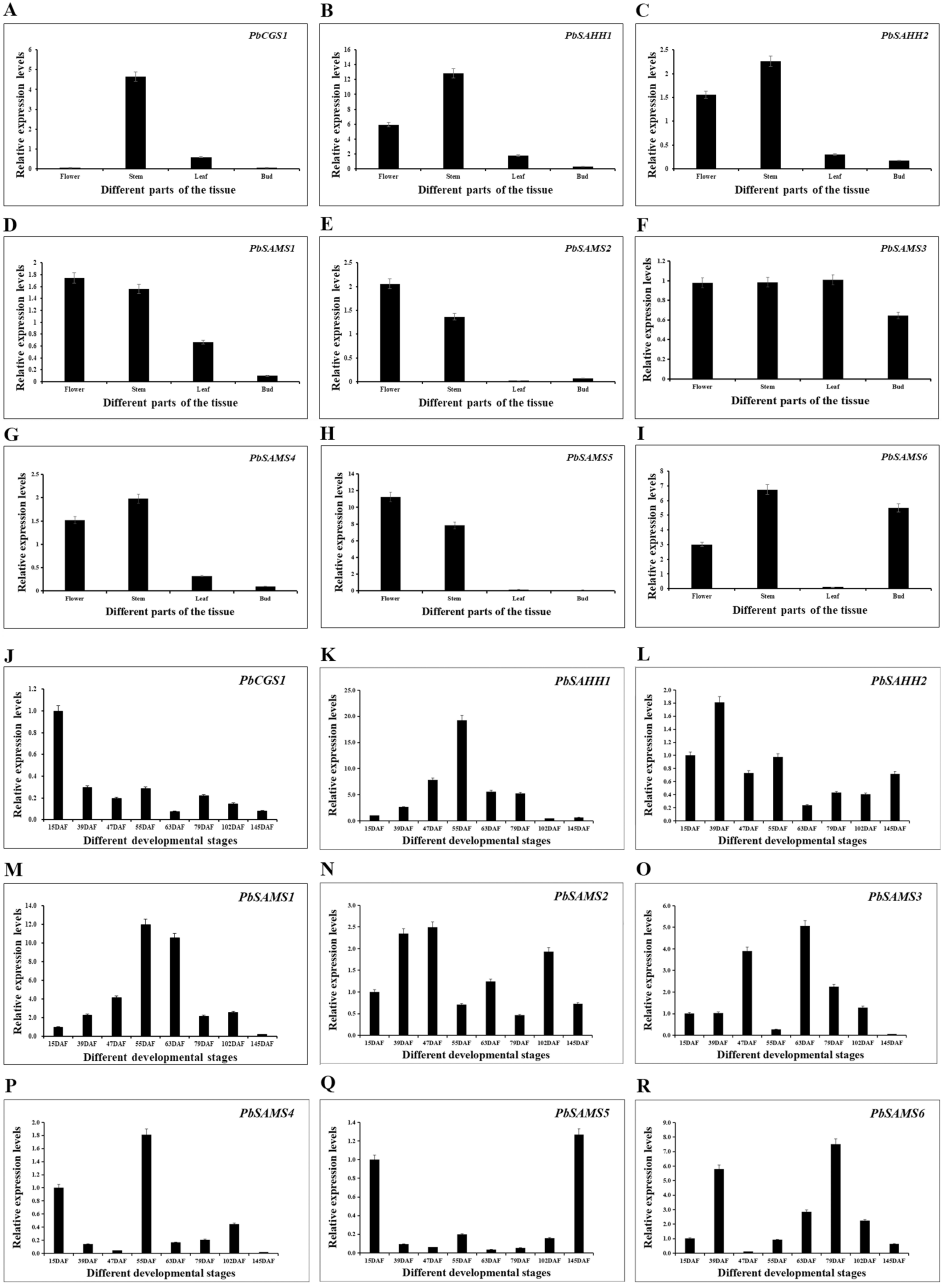
Expression patterns of *PbCGS*, *PbSAHH* and *PbSAMS* genes in different tissues and different fruit development stages in Dangshan Su pear. The expression patterns of *PbCGS*, *PbSAHH* and *PbSAMS* genes in different organs (A-I) of Dangshansu pear. Expression patterns of *PbCGS*, *PbSAHH* and *PbSAMS* genes in Dangshan Su pear at different developmental stages (J-R).

In order to further understand the different functions of the three gene families in pear, we studied the expression patterns of their different tissues (Fig. 13). All members have shown higher expression levels in stems, and three members of *PbSAMS1*, *PbSAMS2* and *PbSAMS5* have the highest level in flowers. *PbCGS1* is only highly expressed in stems. *PbSAHH1*, *PbSAHH2*, *PbSAMS2, PbSAMS4* and *PbSAMS5* are mainly expressed in flowers and stems. *PbSAMS1* is mainly expressed in flowers, stems and leaves. *PbSAMS3* has high expression levels in flowers, stems, leaves and buds. *PbSAMS6* is abundantly expressed in flowers, stems and buds, but hardly expressed in leaves. The content of stone cells is a key factor affecting the quality of pear fruit. The change of lignin content is closely related to the changes in stone cell content. Therefore, we studied the expression patterns of these nine genes in Dangshan Su pear at eight different development stages by qRT-PCR (Fig. 13). The expression levels of *PbCGS1* was the highest in 15 DAF, and the expression levels were low in the remaining seven periods, indicating that they may play an important role in this period. The expression level of *PbSAMS5* was the highest in 15 DAF and 145 DAF, and the expression level was lower in the middle stages of development. *PbSAHH2* and *PbSAMS2* have high expression levels in the early and later stages of development, but moderate expression levels in the middle stages of development. The expression levels of *PbSAMS3*, *PbSAMS4*, and *PbSAMS6* were only highest in the first and middle stages of development but were not high in the other stages. The expression of *PbSAHH1* and *PbSAMS1* is the highest at 55 DAF, which is consistent with the changing trend of lignin content in pear fruits (Cai et al., 2010; Jin et al., 2013), indicating that these two genes may be involved in the synthesis and regulation of lignin in pear fruits.

## Discussion

The size and content of stone cells are the key factors affecting the quality of pear fruit (Cai et al., 2010; Jin et al., 2013). The stone cell is a kind of sclerenchyma cell, which is formed by the continuous deposition of lignin on the secondary wall of parenchyma cells (Yan et al., 2014). Therefore, the formation of a stone cell has a strong correlation with lignin biosynthesis. Through one-carbon metabolism, the three enzymes *CGS*, *SAHH* and *SAMS* control the supply of methyl groups during the methylation reaction (Fig. 1). The CGS controls the synthesis of Met. SAMS catalyzes the synthesis of SAM from ATP and Met. SAHH maintains the SAH level of plants, and SAH competes with SAM binding, thereby inhibiting the activity of methyltransferase. They jointly control the methylation pathway and provide methyl for plant biosynthesis. In the process of G and S lignin biosynthesis, a large number of methyl groups are required. So the screening and identification of these three families are of great significance for an in-depth understanding of pear lignin synthesis and stone cell development regulation.

In this study, we identified 6 *CGS*, 6 *SAHH*, and 28 *SAMS* genes in five Rosaceae species (Table 1). Their isoelectric points are all greater than 7, except for individual ones that are less than 7, indicating that most of the amino acids are acidic. Moreover, most of the *CGS* family members are located in the chloroplast, and most of the *SAHH* and *SAMS* family members are located in the cytoplasm. Phenylalanine ammonia-lyase (PAL) is a key enzyme of lignin biosynthesis in the metabolic pathway of phenylpropane. It is located in the chloroplast and endoplasmic reticulum (Osakabe et al., 2006). Lignin monomer is an important pathway for the metabolism of phenylpropane. Therefore, their biosynthetic pathway is inseparable from the chloroplast and endoplasmic reticulum. So we speculate that three gene families are related to lignin metabolism. Conserved motifs and gene structure may be closely related to the diversity of gene functions (Cao et al., 2018b). We found that the gene structure of the same family members is very similar to the conserved motifs, indicating that their functions are similar, such as *FvSAMS5* and *PmSAMS1*. Their conserved motifs and gene structure (only containing introns, with the same length) are the same. We also found that there are some differences in individual members in each family. Perhaps they have special functions that require special conservative motifs. For example, *PbSAMS6* has motif17, and *FvSAMS2* exists in motif8 and motif20.

Promoters coordinate to regulate gene expression through *cis*-acting elements and trans-acting elements (Soliman & Meyer, 2019). The analysis of this study found that a large number of *cis*-acting elements related to plant hormones, such as CGTCA-motif, TCA-element, ABRE element, and ERE element, are abundant in nine members of pear. These results indicate that these exogenous hormones are widely involved in pear ripening and stress response (Betz, Mccollum & Mayer, 2001). Moreover, we also found some elements involved in biological defense and abiotic stress. For example, TC rich repeats (defense and stress response), MBS (drought-induced), and LTR (low-temperature response), most contain at least one element of MBS or LTR. These results indicate that these members may play an important role in biological defense and abiotic stress. In addition, we only found the light regulatory element (MRE) in *PbSAMS3* and speculated that its expression is also related to light response. We also found that the promoter sequences of *PbSAHH1*, *PbSAMS2*, *PbSAMS5*, and *PbSAMS6* contained AC elements. Studies have shown that this element can bind to MYB to regulate lignin synthesis (Cao et al., 2016), which suggests that they may be involved in lignin synthesis.

Gene expression patterns can provide important clues for exploring gene functions (Budak & Zhang, 2017; Thomas et al., 2018). Previous studies have shown that *CGS* affects the expression of methionine (Hacham, Matityahu & Amir, 2013); SAHH is a key enzyme that maintains the methylation potential of cells (Tanaka et al., 1997; Miller et al., 1994); SAMS catalyzes ATP and Met to synthesize SAM, which is the methylated donor for all organisms in the process of methylation. So far, the role of these genes in pear fruit development is still unclear. Stone cells are an important factor affecting the quality of pear fruit, and lignin is the key to the formation of stone cells. Using RNA-seq (No. SRP070620) and qRT-PCR, the expression patterns of nine genes *PbCGS*/*SAHH*/*SAMS* at different developmental stages of pear fruit were compared. Through RNA-seq data analysis, these nine genes are actively involved in the development of pear fruit. Subsequently, the expression levels of *PbSAMS1* and *PbSAMS3* are similar to the development trend of stone cells in pear fruits. qRT-PCR analysis showed that *PbCGS1* was highly expressed in stem, which indicated that it had a great influence on stem development. All genes have different degrees of expression in flowers, stems, leaves, and buds, and they have different degrees of development in these four parts. We also found that *PbSAHH1* and *PbSAMS1* increased first and then decreased, which is consistent with the development trend of stone cells in pear fruits (Cai et al., 2010; Jin et al., 2013). In *Arabidopsis thaliana*, it has been reported that the reduction of *AtSAMS3* (Shen, Li & Tarczynski, 2002) will lead to the decrease of lignin content in plants, and *AtSAMS3* is homologous with *PbSAMS1*. Combining transcriptome data and qRT-PCR analysis, we speculate *PbSAMS1* may be involved in pear fruit lignin synthesis and stone cell development.

## Conclusions

This research was conducted in five Rosaceae species (*Pyrus bretschneideri*, *Prunus persica*, *Prunus mume*, *Fragaria vesca*, and *Malus domestica*) by using different bioinformatics tools and *in vitro* experiments. However, six *CGS*, six *SAHH*, and 28 *SAMS* genes were identified. The gene structure and conserved motifs of each family are very similar. Promoter analysis found that they contained a large number of *cis*-acting elements related to plant hormones, indicating that hormones play an important role in the regulation of expression patterns during plant growth and development. qRT-PCR analysis showed that among the nine *PbCGS*/*SAHH*/*SAMS* genes were showed abundantly significant expression in flowers and stems except for *PbCGS1*. RNA-seq data and qRT-PCR analysis indicated that *PbSAMS1* may be involved in the regulation of pear stone cell formation and lignin biosynthesis. In conclusion, our observations provide a basis for our understanding of the *PbCGS*/*SAHH*/*SAMS* genes in five Rosaceae plants. In addition, this study revealed their role in pear lignin synthesis and provided basic data for the use of molecular biology techniques to regulate pear lignin synthesis and stone cell development.

## Supplemental Information

10.7717/peerj.13086/supp-1 Supplemental Information 1 Analysis of characteristic domains of *CGS*, *SAHH* and *SAMS*..

10.7717/peerj.13086/supp-2 Supplemental Information 2 Primers of fluorescent quantitative PCR.

10.7717/peerj.13086/supp-3 Supplemental Information 3 Analysis of gene duplication events of *CGS*, *SAHH*, *SAMS* gene family members (They use functional motifs).

10.7717/peerj.13086/supp-4 Supplemental Information 4 Raw data.

## Additional Information and Declarations

### Data Availability

The following information was supplied regarding data availability: The Pyrus bretschneider genome and the gene sequence information of plum (*Prunus mume*) are available at the Genome Database For Rosaceae (https://www.rosaceae.org/), and the sequence information of strawberry (*Fragaria vesca*), peach (*Prunus persica*), and apple (Malus domestica) are available at the Phytozome database (phytozome.jgi.doe.gov/pz/portal.html).
