# Supplementary figures and images for: Whole-genome analysis of CGS, SAHH, SAMS gene families in five Rosaceae species and their expression analysis in Pyrus bretschneideri
Source: PeerJ. 2022 Mar 16;10:e13086. doi: 10.7717/peerj.13086 (PMC8934043; doi:10.7717/peerj.13086)

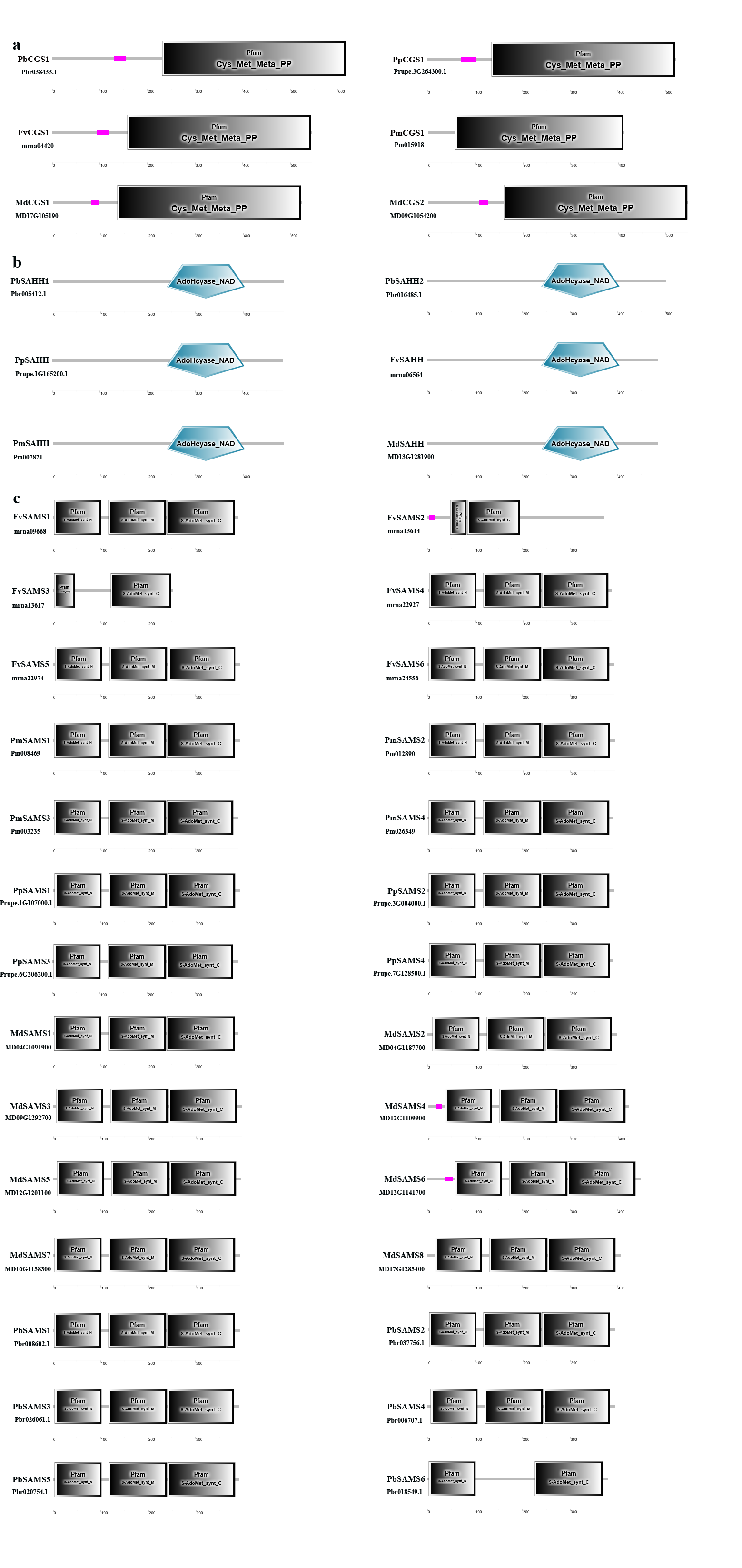

Supplement: Supplemental Information 1 [file peerj-10-13086-s001.png]
