# Supplementary material for: Whole-genome analysis of CGS, SAHH, SAMS gene families in five Rosaceae species and their expression analysis in Pyrus bretschneideri
Source: PeerJ. 2022 Mar 16;10:e13086. doi: 10.7717/peerj.13086 (PMC8934043; doi:10.7717/peerj.13086)
Supplement: Supplemental Information 2 [file peerj-10-13086-s002.docx]

Table S1 Primers of fluorescent quantitative PCR

| **gene** | **Forward primer (5'-3')** |  | **Reverse primer (5'-3')** |  |
| --- | --- | --- | --- | --- |
|  |  |  |  |  |
| ***Tubulin*** | AGAACAAGAACTCGTCCTAC |  | GAACTGCTCGCTCACTCTCC |  |
| ***PbCGS1*** | TTTTGAGATTGATGGAGACTTGACG |  | CTATTAGGCGGGCCACGCTCTATAT |  |
| ***PbSAHH1*** | GCTTGAGTTGTGGAACGAAAGGAAA |  | CTTGTCTCAGTGACGATCCCTCTTC |  |
| ***PbSAHH2*** | CTCGAGTTGTGGAATGAGAGGAAGT |  | TGCTGCAGCAGAAATTATCTCACGC |  |
| ***PbSAMS1*** | GCGGTGGAAGGTTCTTGAAGACAGC |  | GCAGGGGCGGTATTTGATCCATCTC |  |
| ***PbSAMS2*** | GAGCCTCTGTCAGTGTTCGTCGACT |  | AAGAGCCACCATTAAAGCCTTCAAG | |
| ***PbSAMS3*** | GCTTGTCGGAATGGAAGACACCTTC |  | CCAACATCATCTGAAACAAATCCAA |  |
| ***PbSAMS4*** | TCTTACAAGCATGGCAGAGTCGGCA |  | CCGAAACAAATCCAATTGTACGGCA | |
| ***PbSAMS5*** | TATGCTATTGGCGTCCCAGAACCCC |  | GAACTGCGGCTACGAGCTAGTGTGT | |
| ***PbSAMS6*** | GTCCCAATTCGCGTCCACACCGTCC |  | CAATGGCCTTTGCAGCCTGCCTCCC |  |
