# Supplementary material for: Whole-genome analysis of CGS, SAHH, SAMS gene families in five Rosaceae species and their expression analysis in Pyrus bretschneideri
Source: PeerJ. 2022 Mar 16;10:e13086. doi: 10.7717/peerj.13086 (PMC8934043; doi:10.7717/peerj.13086)
Supplement: Supplemental Information 3 [file peerj-10-13086-s003.docx]

Table S2 Analysis of gene duplication events of *CGS*, *SAHH*, *SAMS* gene family members (They use functional motifs)

| **Duplicated genes** | **Ka** | **Ks** | **Ka/Ks** | **Duplicated type** |
| --- | --- | --- | --- | --- |
| **(*PbSAMS2*)*Pbr037756.1/*(*PbSAMS6*)*Pbr018549.1*** | **0.0448** | **0.2899** | **0.1545** | **Segmental Duplication** |
| **(*PbSAMS3*)*Pbr026061.1/*(*PbSAMS4*)*Pbr006707.1*** | **0.0147** | **0.1999** | **0.0735** | **Segmental Duplication** |
| **(*FvSAMS5*)*mrna22974/*(*FvSAMS6*)*mrna24556*** | **0.0419** | **1.0464** | **0.0400** | **Segmental Duplication** |
| **(*MdSAMS2*)*MD04G1187700/*(*MdSAMS5*)*MD12G1201100*** | **0.0171** | **0.2363** | **0.0724** | **Segmental Duplication** |
| **(*MdSAMS3*)*MD09G1292700/*(*MdSAMS8*)*MD17G1283400*** | **0.0024** | **0.2180** | **0.0110** | **Segmental Duplication** |
